# Knowledge, attitudes, and practices of family caregivers for patients with cerebral infarction toward home-based care

**DOI:** 10.3389/fpubh.2024.1436423

**Published:** 2024-08-20

**Authors:** Zhipeng Chen, Xiaohua Zhou, Lu Jiang, Chunmei Song, Shufang Wang, Huilan Zhao, Jianping Liu, Xiangxiang Ma, Jia Yu

**Affiliations:** Department of Neurology, The Yancheng School of Clinical Medicine of Nanjing Medical University, Yancheng Third People's Hospital, Yancheng, China

**Keywords:** knowledge, attitudes, practices, cerebral infarction, cross-sectional study, home care services

## Abstract

**Objective:**

This study aimed to assess the knowledge, attitudes, and practices (KAP) among family caregivers of patients with cerebral infarction toward home-based care.

**Methods:**

This web-based cross-sectional study was conducted between October 2023 and February 2024 at Yancheng Third People’s Hospital. A self-designed questionnaire was developed to collect demographic information, and assess the KAP among family caregivers of patients with cerebral infarction toward home-based care.

**Results:**

A total of 761 questionnaires were included in the study. Among the participants, 453 (59.53%) were female, and 548 (72.01%) lived with the patients. The mean knowledge, attitudes and practices scores were 6.67 ± 1.73 (possible range: 0–9), 32.95 ± 2.46 (possible range: 9–45), and 28.64 ± 4.39 (possible range: 8–40), respectively. Path analysis showed the direct effect of knowledge on both attitudes (*β* = 0.885, *p* < 0.001) and practices (*β* = 1.295, *p* < 0.001), as well as of attitudes on practices (*β* = 0.838, *p* < 0.001).

**Conclusion:**

Family caregivers of patients with cerebral infarction have sufficient knowledge, positive attitudes and proactive practices toward home-based care. However, they still exhibit deficiencies in certain aspects of knowledge, attitudes, and practice. Developing personalized educational strategies may be instrumental in enhancing family caregivers’ knowledge of home-based care. This, in turn, could improve their attitudes and elevate their practice levels.

## Introduction

The escalating global burden of strokes, as reported by the World Stroke Organization, underscores an urgent health crisis, with over 12 million new cases annually and more than 100 million individuals currently living with the condition (1). Within this context, cerebral infarction, the predominant form of cerebrovascular disease, has been on the rise, partly due to demographic shifts toward an older population (2). This condition, which is primarily categorized into hemorrhagic and ischemic types, represents about 80% of all stroke occurrences (3). Moreover, cerebral infarction is distinguished by its severe outcomes, being the second leading cause of death from diseases worldwide and resulting in serious long-term impairments such as memory disorders and paralysis among survivors (4).

Based on epidemiological data from 2013, the incidence and mortality rates of cerebral infarction in China significantly exceeded the global average, with reported incidences of 42% in Chinese men and 37% in Chinese women (5). A 2019 study published in the Lancet identified cerebral stroke as the leading cause of death in China over the period from 1990 to 2017 (6). In recent years, the aging population in China has contributed to an increase in cerebral infarction cases. Although mortality rates have declined compared to 10 years ago, the disability rate associated with this condition remains alarmingly high. Approximately 75% of those affected by cerebral infarction suffer from neurological deficits leading to disability, and 40% experience severe disability (7, 8).

In response to these daunting challenges, the significance of home healthcare, also known as in-home care, social care, or domiciliary care, has been increasingly recognized within the healthcare paradigm (9). Following acute and rehabilitation treatment phases, a substantial majority of stroke survivors, approximately 80%, opt for returning home. Here, they rely heavily on family caregivers for the bulk of their ongoing care needs, with these caregivers dedicating an average of 35 h per week in providing care during the first year after a stroke (10).

The Knowledge-Attitude-Practice (KAP) theory occupies a central position in influencing human health behaviors (11). It is frequently utilized in conjunction with the KAP questionnaire to thoroughly assess the knowledge, attitudes, and practices of the target population within the healthcare sector (12). This approach also evaluates the demand for and level of acceptance of pertinent information. The model, which is vital to health literacy, is founded on the essential belief that knowledge positively affects attitudes, which, in turn, influence individual behaviors (13). Given that the vast majority of stroke survivors opt to continue their rehabilitation at home, family caregivers play a crucial role in the daily care of patients with cerebral infarction. They have a direct impact on the health recovery and quality of life of the patients. Investigating this group can help us understand their needs, knowledge gaps, and challenges faced. This, in turn, can lead to the provision of targeted training and resources to enhance the quality of care. Moreover, while providing care, family caregivers often endure significant emotional and financial stress. Research can uncover their needs and challenges, offering them the necessary support and resources to alleviate their burden and improve their caregiving capabilities.

Despite its significance, there is a lack of research in this field in China (14, 15). Therefore, this study aimed to assess the KAP of family caregivers of patients with cerebral infarction regarding home-based care.

## Methods

### Study design and participants

This cross-sectional survey was conducted between October 2023 to February 2024 at Yancheng Third People’s Hospital. The study was approved by the Ethics Committee of Yancheng Third People’s Hospital (Approval No. 2023–61).

The calculation of sample size was as follows (16):


$$n={\left(\frac{Z_{1-\frac{\alpha }{2}}}{\delta }\right)}^{2}\times p\times \left(1-p\right)$$

Here, n represents the sample size, with p assumed to be 0.5 to ensure the maximum sample size. The type I error, denoted as α, was set to 0.05. Given this, ${Z\;}_{1-\frac{\alpha }{2}}\;=1.96.$ Assuming an effective questionnaire recovery rate of 80%, the final target was to collect at least 480 completed questionnaires.

Convenience sampling was employed to recruit family caregivers of patients with cerebral infarction. Inclusion criteria encompassed: (1) being a primary caregiver of a patient diagnosed with cerebral infarction, (2) aged 18 years or older, (3) proficient in Mandarin. Exclusion criteria consisted of: (1) caregivers of patients with concurrent severe medical conditions, such as terminal illnesses, (2) caregivers unable to provide informed consent, (3) caregivers with a history of psychiatric disorders or cognitive impairment affecting their ability to participate effectively.

### Questionnaire

The questionnaire was formulated based on pertinent literature (17–19). The initial iteration of the study underwent refinement through multiple revisions, incorporating feedback from experts including WSF (Associate Chief Nurse, 31 years of experience in neurology nursing), PXQ (Associate Chief Nurse, 30 years of experience in neurology nursing), and LXM (33 years of experience in neurology nursing). Following this iterative process, a preliminary trial was conducted on a limited scale (*n* = 42), yielding a Cronbach’s alpha coefficient value of 0.834, indicating good internal consistency.

The final questionnaire was in Chinese, and comprised four dimensions: demographic information, knowledge, attitudes, and practices. The demographic section consisted of 19 items, while the knowledge, attitudes, and practices dimensions included 11, 9, and 9 items, respectively. Notably, Questions K4 and K11 within the knowledge dimension were designed as trap questions. These questions were intentionally designed with exactly opposite meanings, thereby identifying respondents with logical inconsistencies. Participants selecting contradictory responses for both questions were excluded from the survey. Consequently, Questions K4 and K11 were excluded from subsequent statistical analyses. Scoring for the knowledge dimension assigned 1 point for correct answers and 0 points otherwise, resulting in a potential score range of 0–9. Attitudes were assessed using a five-point Likert scale ranging from very positive (5 points) to very negative (1 point), with a potential score range of 9 to 45. Similarly, practices were rated on a five-point Likert scale, ranging from very consistent (5 points) to very inconsistent (1 point). Question P4 served as a descriptive item and was not factored into the overall score calculation. Consequently, the potential score range for practices was 8 to 40. Sufficient knowledge, positive attitudes, and proactive practices were defined as scores exceeding 70% of the maximum achievable score in each respective section (20).

The data were gathered via an online questionnaire administered through Sojump.^1^ Upon initiating the electronic questionnaire, participants were prompted to affirm their consent by selecting the option “I agree to participate in this study” before proceeding to respond to the inquiries. All data collection procedures were carried out anonymously. To mitigate the possibility of duplicate submissions, IP restrictions were enforced, permitting completion of the survey only once from each unique IP address.

### Statistical analysis

Data analysis was conducted using STATA 17.0 (Stata Corporation, College Station, TX, USA). Continuous data are presented as means and standard deviations (SD), while categorical data are expressed as n (%). Continuous variables underwent a normality test, with the t-test for normally distributed data and the Wilcoxon Mann–Whitney test for non-normally distributed data when comparing two groups. For three or more groups with normally distributed continuous variables and uniform variance, ANOVA was used for comparisons, while the Kruskal-Wallis test was employed for non-normally distributed data. Path analysis of the relationships between knowledge, attitudes, and practices was conducted using AMOS 24.0 (IBM, NY, United States). The path analysis examined the following primary hypotheses: (1) Knowledge exerted direct effects on attitudes, (2) Knowledge exerted direct effects on practices, and (3) Attitudes exerted direct effects on practices. A two-sided *p*-value less than 0.05 was considered statistically significant.

## Results

Initially, 811 questionnaires were collected. Of these, 50 questionnaires were excluded due to incorrect answers to the trap questions. Consequently, a total of 761 questionnaires were ultimately included in the study, with an overall validity rate of the questionnaires of 93.83%.

Thus, a total of 761 participants enrolled in this study. Among the participants, 453 (59.53%) were female, 417 (54.80%) were aged 51–70 years, 475 (62.42%) had junior high school education or below, 438 (57.56%) had an average monthly household income of 2,000–5,000 CNY, and 548 (72.01%) lived with the patients. Regarding the patients under their care, 443 (58.21%) of the patients were male, 429 (56.37%) were aged 71–90 years, 318 (41.79%) suffered from cerebral infarction for less than a month, 589 (77.4%) also suffered from hypertension, 319 (41.92%) were disabled, and 424 (55.72%) preferred a high salt/fat diet. The mean knowledge, attitudes and practices scores were 6.67 ± 1.73, 32.95 ± 2.46 and 28.64 ± 4.39, respectively (Table 1).

**Table 1:** Demographic characteristics.

	*n* (%)	Knowledge		Attitudes		Practices	
		mean ± SD	*p*	mean ± SD	*p*	mean ± SD	*p*
*N* = 761							
Total Score		6.67 ± 1.73		32.95 ± 2.46		28.64 ± 4.39	
Gender			0.307		0.035		0.034
Male	308 (40.47)	6.59 ± 1.70		33.18 ± 2.52		28.23 ± 4.72	
Female	453 (59.53)	6.72 ± 1.75		32.80 ± 2.41		28.92 ± 4.13	
Age			<0.001		0.005		0.017
18–30 years old	16 (2.10)	6.38 ± 2.16		34.19 ± 3.37		28.94 ± 3.92	
31–50 years old	217 (28.52)	6.97 ± 1.54		33.11 ± 2.65		28.32 ± 4.59	
51–70 years old	417 (54.80)	6.73 ± 1.70		33.00 ± 2.25		29.05 ± 4.23	
≥ 71 years old	111 (14.59)	5.91 ± 1.93		32.31 ± 2.60		27.68 ± 4.46	
Residence			<0.001		0.001		<0.001
Rural	306 (40.21)	6.40 ± 1.85		32.60 ± 2.24		27.95 ± 4.34	
Urban	455 (59.79)	6.85 ± 1.63		33.19 ± 2.57		29.11 ± 4.36	
Marital status			0.306		0.564		0.882
Unmarried/Divorced/Other	27 (3.55)	6.33 ± 2.04		33.22 ± 3.04		28.52 ± 4.36	
Married	734 (96.45)	6.68 ± 1.72		32.94 ± 2.44		28.65 ± 4.39	
Education			<0.001		<0.001		<0.001
Junior high school and below	475 (62.42)	6.41 ± 1.81		32.55 ± 2.26		28.08 ± 4.45	
High school and technical school	214 (28.12)	7.04 ± 1.45		33.29 ± 2.29		29.47 ± 3.99	
College and above	72 (9.46)	7.26 ± 1.67		34.60 ± 3.29		29.9 ± 4.46	
Average Monthly Household Income (CNY)			<0.001		<0.001		0.001
< 2000	124 (16.29)	6.50 ± 1.77		32.41 ± 2.25		28.66 ± 4.03	
2000–5,000	438 (57.56)	6.57 ± 1.77		32.76 ± 2.31		28.42 ± 4.46	
5,000–10,000	179 (23.52)	6.88 ± 1.59		33.52 ± 2.44		28.75 ± 4.38	
> 10,000	20 (2.63)	8.10 ± 1.07		35.60 ± 4.16		32.35 ± 3.45	
Occupation			<0.001		0.110		<0.001
Formal employment/part-time/self-employment	394 (51.77)	6.75 ± 1.68		33.03 ± 2.56		28.34 ± 4.55	
Unemployed	29 (3.81)	7.07 ± 1.33		33.48 ± 2.16		29.69 ± 2.71	
Retired	197 (25.89)	6.92 ± 1.56		33.04 ± 2.30		29.72 ± 3.96	
Other	141 (18.53)	6.01 ± 2.02		32.53 ± 2.44		27.77 ± 4.46	
Relationship			0.051		0.306		0.203
Spouse of the patient	346 (45.47)	6.53 ± 1.76		32.79 ± 2.37		28.81 ± 4.15	
Children of the patient	370 (48.62)	6.74 ± 1.69		33.06 ± 2.51		28.42 ± 4.64	
Other family relationship	31 (4.07)	7.00 ± 1.86		33.48 ± 2.91		28.55 ± 3.33	
Employment/other	14 (1.84)	7.57 ± 1.83		33.07 ± 2.23		30.71 ± 4.91	
Living with the patient			0.415		0.160		0.002
Yes	548 (72.01)	6.64 ± 1.73		32.88 ± 2.37		28.95 ± 4.2	
No	213 (27.99)	6.75 ± 1.75		33.15 ± 2.68		27.85 ± 4.75	
Patient’s Gender			0.855		0.103		0.221
Male	443 (58.21)	6.66 ± 1.75		32.83 ± 2.38		28.48 ± 4.44	
Female	318 (41.79)	6.68 ± 1.72		33.13 ± 2.56		28.87 ± 4.31	
Patient’s Age			0.082		0.166		0.002
31–50 years old	30 (3.94)	7.10 ± 1.73		32.67 ± 2.60		27.27 ± 4.31	
51–70 years old	302 (39.68)	6.78 ± 1.59		33.16 ± 2.31		29.3 ± 4.08	
≥ 71 years old	429 (56.37)	6.56 ± 1.82		32.83 ± 2.55		28.28 ± 4.54	
Medical Insurance			<0.001		0.837		<0.001
No medical insurance	6 (0.79)	8.17 ± 0.75		33.33 ± 1.63		27.33 ± 5.68	
Only basic medical insurance	638 (83.84)	6.57 ± 1.78		32.97 ± 2.51		28.29 ± 4.43	
Basic medical insurance and other commercial insurance	117 (15.37)	7.15 ± 1.33		32.85 ± 2.24		30.62 ± 3.46	
Patients’ comorbidities (Multiple choices)							
Hypertension (or long-term use of antihypertensive drugs)	589 (77.4)	6.76 ± 1.70		32.89 ± 2.43		28.72 ± 4.32	
Hyperlipidemia	72 (9.46)	6.92 ± 1.91		33.54 ± 3.02		29.01 ± 5.13	
Diabetes	245 (32.19)	6.94 ± 1.68		33.02 ± 2.38		29.04 ± 4.15	
Malignant tumor	8 (1.05)	5.88 ± 1.55		33.88 ± 3.87		26.38 ± 6.14	
Other	116 (15.24)	6.26 ± 1.97		33.14 ± 2.57		28.18 ± 5.11	
None of the above	65 (8.54)	6.32 ± 1.74		33.57 ± 2.45		28.09 ± 5.28	
Patient’s Smoking Habit			0.347		0.030		0.055
Yes	253 (33.25)	6.58 ± 1.86		32.68 ± 2.32		28.21 ± 4.40	
No	508 (66.75)	6.71 ± 1.67		33.09 ± 2.52		28.86 ± 4.37	
Patient’s Drinking Habit			0.990		0.714		0.366
Yes	191 (25.1)	6.67 ± 1.73		33.01 ± 2.62		28.39 ± 4.74	
No	570 (74.9)	6.67 ± 1.74		32.94 ± 2.41		28.72 ± 4.26	
Disability status of the patient			<0.001		0.103		0.013
Yes	319 (41.92)	7.03 ± 1.53		33.13 ± 2.45		29.10 ± 4.00	
No	442 (58.08)	6.41 ± 1.83		32.83 ± 2.46		28.31 ± 4.62	
Does the patient prefer a high-salt/high-fat diet?			0.464		0.017		0.689
Yes	424 (55.72)	6.71 ± 1.69		32.76 ± 2.44		28.70 ± 4.29	
No	337 (44.28)	6.62 ± 1.79		33.19 ± 2.47		28.57 ± 4.51	
Patient’s Exercise Frequency			<0.001		0.056		<0.001
1 ~ 2 times a week	81 (10.64)	5.77 ± 2.11		33.28 ± 3.44		27.09 ± 5.62	
3 ~ 4 times a week	113 (14.85)	6.88 ± 1.77		32.70 ± 2.00		28.99 ± 3.36	
5 ~ 6 times a week	173 (22.73)	7.20 ± 1.31		32.56 ± 1.90		30.05 ± 2.48	
Every day	292 (38.37)	6.59 ± 1.69		33.12 ± 2.51		28.38 ± 4.57	
Never	102 (13.4)	6.47 ± 1.79		33.17 ± 2.60		27.84 ± 5.49	
If “Never” was not selected in the previous question, what type of exercise does the patient usually engage in? (*n* = 659)			0.023		<0.001		0.008
Aerobic exercise (such as walking, cycling, jogging, tai chi, etc.)	524 (68.86)	6.61 ± 1.78		32.98 ± 2.49		28.65 ± 4.35	
Resistance training (such as strength training, leg raises, push-ups, dumbbells, etc.)	134 (17.61)	7.03 ± 1.42		32.61 ± 2.01		29.12 ± 3.31	
Both of the above	1 (0.13)	9.00		44.00		40.00	
Duration of the patient’s illness			0.001		0.001		0.049
Less than 1 month	318 (41.79)	6.74 ± 1.66		32.81 ± 2.16		28.78 ± 4.20	
1 ~ 6 months	93 (12.22)	7.08 ± 1.45		33.60 ± 2.34		29.27 ± 3.67	
6 ~ 12 months	117 (15.37)	6.55 ± 1.65		32.32 ± 2.36		28.49 ± 4.27	
12 ~ 18 months	24 (3.15)	7.00 ± 1.35		32.71 ± 1.68		29.75 ± 3.66	
18 ~ 24 months	49 (6.44)	7.04 ± 1.41		32.94 ± 2.43		29.22 ± 3.89	
>24 months	160 (21.02)	6.23 ± 2.11		33.38 ± 3.07		27.77 ± 5.28	

The knowledge assessment highlighted that the highest correctness rates were 93.82% for recognizing the importance of maintaining hygiene, diet, exercise, and sleep in family care for ischemic stroke patients (K9), and 91.72% for the necessity of psychological counseling (K7). In contrast, the lowest correctness rates were 37.06% for common treatment modalities (K3), and 62.94% for accurately defining ischemic stroke (K1). Furthermore, 80.16% acknowledged the importance of home care in enhancing patient quality of life (A5), and 76.61% emphasized the need for patience and guidance in such care (P8). Neutral attitudes were recorded with 54.93% unsure whether home care leads to irritability or anxiety (A4), and 46.91% on the effectiveness of home care’s role (A7). Home care practices focused on improving treatment adherence (74.24%, P8), sharing caregiving experiences (68.46%, P2), acquiring care-related knowledge (48.23%, P1), and providing psychological support (44.15%, P4). Detailed distributions of these findings are presented in Tables 2–4.

**Table 2:** Knowledge.

	Correctness *N* (%)
K1. Ischemic stroke refers to the localized ischemia and hypoxic necrosis of brain tissue caused by cerebrovascular insufficiency. It is a common cerebrovascular disease clinically known as “ischemic stroke.” (True)	479 (62.94)
K2. Sequelae of ischemic stroke include speech impairment, limb paralysis, and others. (True)	644 (84.62)
K3. Common treatment modalities for ischemic stroke include medication, thrombolysis, and thrombectomy. (True)	282 (37.06)
K5. Family care for ischemic stroke patients encompasses comprehensive interventions across multiple dimensions such as physiology, psychology, social interactions, and health behaviors. (True)	660 (86.73)
K6. Physiological care for ischemic stroke patients involves limb rehabilitation training (assistance with turning over/walking, etc.), language rehabilitation training (tongue movement/swallowing function training, etc.), and activities of daily living (self-dressing, hair combing, grip training, etc.). (True)	559 (73.46)
K7. Psychological counseling is essential for ischemic stroke patients to alleviate anxiety and other emotional distress. (True)	698 (91.72)
K8. Health monitoring for ischemic stroke patients, such as blood pressure and blood glucose level measurements, falls outside the scope of patient disease home care. (False)	490 (64.39)
K9. Family care for ischemic stroke patients should include maintaining personal hygiene, adopting a healthy diet, and cultivating good exercise and sleep habits. (True)	714 (93.82)
K10. Patients with ischemic stroke and hemiplegia have a weakened immune system, necessitating particular attention to preventive measures against infectious diseases such as pulmonary infections. (True)	549 (72.14)

**Table 3:** Attitudes.

	Strongly agree *N* (%)	Agree *N* (%)	Neutral *N* (%)	Disagree *N* (%)	Strongly disagree *N* (%)
A1. Caregivers of ischemic stroke patients play a crucial role in the patient’s disease rehabilitation process. (Positive)	158 (20.76)	548 (72.01)	55 (7.23)	0	0
A2. You are willing to obtain various information related to home care for ischemic stroke patients through various channels such as the internet, television, books, etc. (Positive)	25 (3.28)	435 (57.16)	292 (38.37)	9 (1.18)	0
A3. If the hospital provides in-hospital education related to home care for ischemic stroke patients, you are very willing to participate. (Positive)	18 (2.37)	387 (50.85)	350 (45.99)	6 (0.79)	0
A4. You believe that engaging in the patient’s home care will consume a significant amount of your energy, and sometimes caregiving may make you feel irritable or anxious. (Negative)	8 (1.05)	163 (21.42)	418 (54.93)	167 (21.94)	5 (0.66)
A5. You recognize the importance of home care in improving the quality of life for ischemic stroke patients. (Positive)	73 (9.59)	610 (80.16)	78 (10.25)	0	0
A6. Home care for ischemic stroke patients not only requires attention to the patient’s physical condition but also involves crucial psychological counseling for the patient. (Positive)	34 (4.47)	570 (74.90)	156 (20.50)	1 (0.13)	0
A7. You believe that the role of home care is relatively limited, and the majority of the patient’s recovery depends on hospital treatment. (Negative)	35 (4.60)	252 (33.11)	357 (46.91)	117 (15.37)	0
A8. Patient education and guidance, helping patients develop good habits, gradually restoring some physical functions, and enhancing the patient’s self-efficacy are particularly important in home-care. (Positive)	70 (9.20)	583 (76.61)	107 (14.06)	1 (0.13)	0
A9. You believe that caregivers of ischemic stroke patients actively cooperating with the doctor’s treatment plan, increasing patient compliance, and timely communication with medical staff are crucial for disease improvement. (Positive)	151 (19.84)	564 (74.11)	46 (6.04)	0	0

**Table 4:** Practices.

	Always *N* (%)	Often *N* (%)	Sometimes *N* (%)	Occasionally *N* (%)	Never *N* (%)
P1. The frequency at which you actively seek knowledge related to home care for ischemic stroke patients through various channels (such as books, the internet, communication with doctors, etc.).	17 (2.23)	133 (17.48)	367 (48.23)	160 (21.02)	84 (11.04)
P3. The frequency at which you guide patients in exercise rehabilitation and maintaining good lifestyle habits during home care.	33 (4.34)	284 (37.32)	269 (35.35)	98 (12.88)	77 (10.12)
P5. The frequency at which you assist patients in maintaining personal and environmental hygiene (such as assisting with changing clean clothes, maintaining oral hygiene, keeping the patient’s rest area clean, etc.).	83 (10.91)	395 (51.91)	185 (24.31)	72 (9.46)	26 (3.42)
P6. The frequency at which you provide nutritional support (such as preparing light and healthy meals) during home care.	111 (14.59)	366 (48.09)	193 (25.36)	70 (9.20)	21 (2.76)
	Completely consistent *N* (%)	Consistent *N* (%)	Not necessarily *N* (%)	Inconsistent *N* (%)	Completely inconsistent *N* (%)
P2. The frequency at which you communicate caregiving experiences with other caregivers during the patient’s home care.	46 (6.04)	521 (68.46)	177 (23.26)	17 (2.23)	0
P7. The frequency at which you plan to accompany the patient to hospital follow-up visits regularly, to monitor the patient’s recovery progress.	93 (12.22)	508 (66.75)	117 (15.37)	40 (5.26)	3 (0.39)
P8. The extent to which you focus on improving the patient’s compliance with treatment and enhancing the patient’s self-management efficacy during home care.	62 (8.15)	565 (74.24)	107 (14.06)	26 (3.42)	1 (0.13)
P9. The extent to which you consciously pay attention to the patient’s requests and various signs during caregiving, and monitor the patient’s blood pressure, blood sugar, and other indicators, providing comprehensive care for the patient.	170 (22.34)	466 (61.24)	97 (12.75)	27 (3.55)	1 (0.13)

P4. The frequency at which you provide emotional support and conduct psychological intervention for ischemic stroke patients during home care.	
Always *N* (%)	22 (2.89)
Often *N* (%)	253 (33.25)
Sometimes *N* (%)	336 (44.15)
Occasionally *N* (%)	87 (11.43)
Never *N* (%)	27 (3.55)
The patient’s overall psychological state is good; therefore, psychological intervention was not conducted. *N* (%)	15 (1.97)
The patient has lost basic responsiveness or is experiencing confusion, so psychological intervention was not undertaken. *N* (%)	21 (2.76)

In the correlation analyses, significant positive correlations were found between knowledge and attitude (*r* = 0.339, *p* = 0.002), knowledge and practice (*r* = 0.546, *p* < 0.001), and attitude and practice (*r* = 0.416, *p* < 0.001), respectively (Supplementary Table S1).

458 (60.18%), 569 (74.64%), and 545 (71.62%) of the participants had sufficient knowledge, positive attitudes and positive practices (Supplementary Table S2). Multivariate logistic regression showed that high school and technical school education (OR = 1.705, 95% CI: [1.124, 2.586], *p* = 0.012), with average monthly household income of more than 10,000 Yuan (OR = 5.830, 95% CI: [1.060, 32.072], *p* = 0.043), hiring to care for patients (OR = 7.856, 95% CI: [1.436, 42.986], *p* = 0.017), in which the patient has a disability (OR = 3.580, 95% CI: [2.358, 5.436], *p* < 0.001), and more than 2 times a week of exercise by patients (OR > 1, *p* < 0.05) were independently associated with adequate knowledge (Supplementary Table S3). Meanwhile, knowledge score (OR = 1.420, 95% CI: [1.274, 1.583], *p* < 0.001), high school and technical school education (OR = 1.680, 95% CI: [1.061, 2.661], *p* = 0.027), college education and above (OR = 2.594, 95% CI: [1.084, 6.208], *p* = 0.032), female patients (OR = 1.651, 95% CI: [1.136, 2.398], *p* = 0.009), and 1–6 months of illness (OR = 2.169, 95% CI: [1.091, 4.312], *p* = 0.027) were independently associated with favorable attitudes. While, 6–12 months of illness (OR = 0.513, 95% CI: [0.315, 0.835], *p* = 0.007) was independently associated with poor attitudes (Supplementary Table S4). Furthermore, knowledge score (OR = 1.756, 95% CI: [1.541, 2.001], *p* < 0.001), attitude score (OR = 1.203, 95% CI: [1.098, 1.318], *p* < 0.001), living with the patient (OR = 2.046, 95% CI: [1.314, 3.187], *p* = 0.002), 3–4 times a week of exercise by patients (OR = 2.792, 95% CI: [1.383, 5.637], *p* = 0.004), and 5–6 times a week of exercise by patients (OR = 5.437, 95% CI: [2.635, 11.22], *p* < 0.001) were independently associated with positive practices. While, more than 24 months of illness (OR = 0.581, 95% CI: [0.347, 0.973], *p* = 0.039) was independently associated with negative practices (Supplementary Table S5).

**Figure 1:**
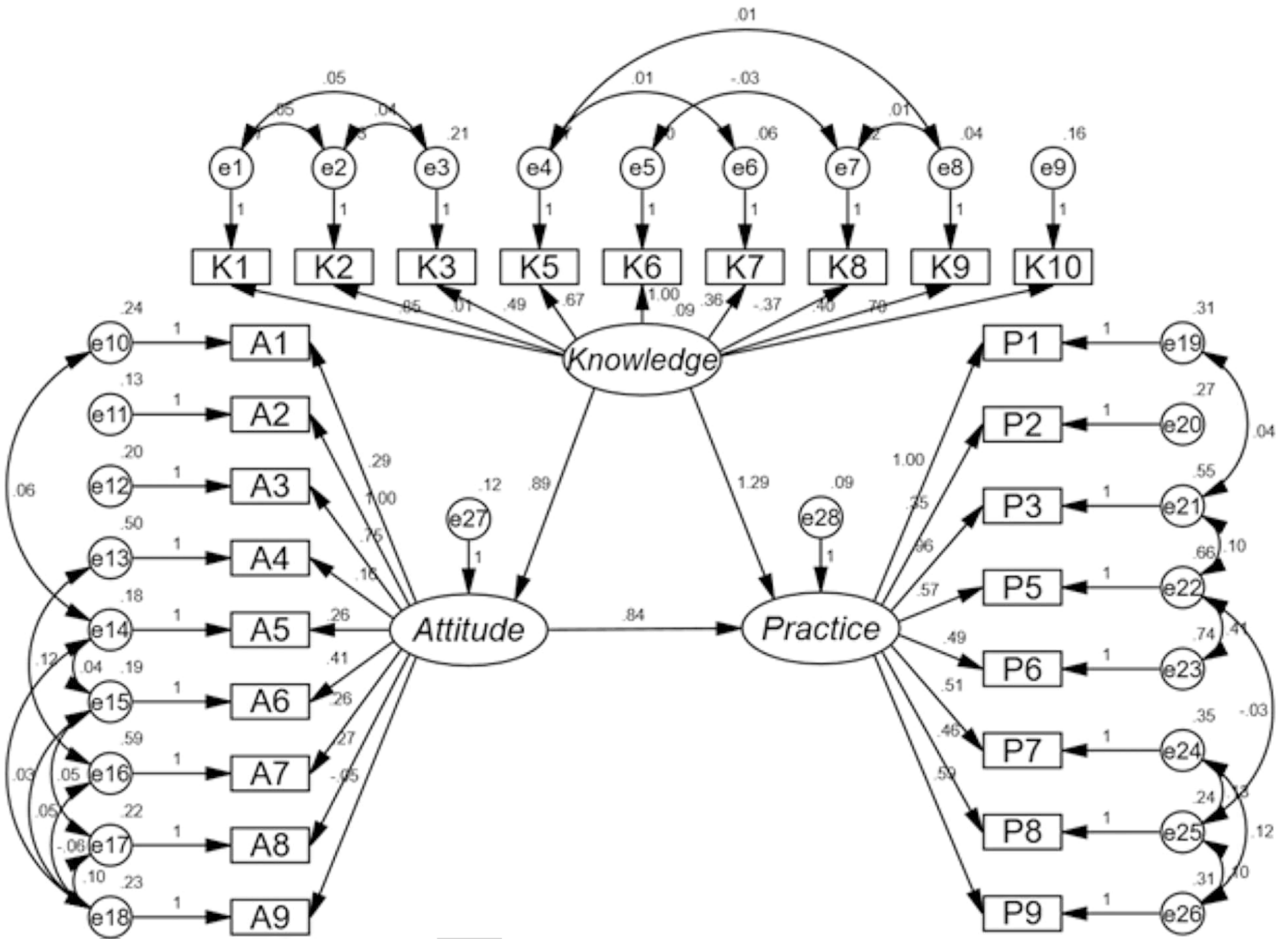
The KAP structural equation model.

The fit of the path analysis model yielded good indices demonstrating good model fit (Supplementary Table S7), and the results showed that the direct effect of knowledge on both attitudes (*β* = 0.885, *p* < 0.001) and practices (*β* = 1.295, *p* < 0.001), as well as of attitudes on practices (*β* = 0.838, *p* < 0.001) (Supplementary Table S6 and Figure 1).

## Discussion

Family caregivers of patients with cerebral infarction have sufficient knowledge, positive attitudes and proactive practices toward home-based care. However, they still exhibit deficiencies in certain aspects of knowledge, attitudes, and practice.

Previous studies caregivers in stroke management reveals alignments with the findings on home-based care. For instance, caregivers in rural areas exhibited less knowledge about stroke complications, which aligns with observation of deficiencies in certain knowledge aspects despite generally proactive practices (14). Similarly, the study on poststroke depression indicated an overall insufficient knowledge among caregivers, yet it demonstrated a positive correlation between knowledge and both attitude and practice, supporting the premise that enhanced knowledge positively impacts caregiving behavior (21). Contrasting this, the research focusing on the needs and competences of younger, non-white female carers highlights significant challenges post-discharge, emphasizing the necessity for targeted educational interventions (22). This notion of demographic-specific educational needs is further corroborated by findings on caregivers’ limited understanding of stroke etiology and recovery expectations in a rehabilitation setting, underlining the ongoing requirement for comprehensive education to bridge knowledge gaps (23). Together, these studies emphasize the critical role of tailored educational strategies that not only address general knowledge but also cater to specific caregiver demographics to enhance overall care efficacy.

The results from both inter-group comparisons and multivariate logistic regression analyses offer valuable insights into the factors influencing the KAP of family caregivers of patients with cerebral infarction. Educational attainment emerges as a significant factor in both sets of analyses. Higher education levels consistently correlate with higher KAP scores among caregivers. This finding aligns with prior research, highlighting the importance of education in healthcare decision-making and caregiving competence (14). Caregivers with higher education levels may possess better health literacy, enabling them to access and comprehend relevant information, ultimately leading to more informed caregiving practices. Similarly, socioeconomic status, as indicated by average monthly household income, emerges as a strong predictor of caregiving KAP. Higher income levels are consistently associated with superior KAP scores among caregivers. This finding underscores the role of financial resources in caregiving, as caregivers from higher-income households may have greater access to support services, healthcare resources, and caregiving aids (24, 25). Furthermore, patient disability status consistently emerges as a significant predictor of caregiving KAP. Caregivers of disabled patients demonstrate higher KAP scores across various dimensions. This finding suggests that the level of patient dependency significantly influences caregiver knowledge, attitudes, and practices. Caregivers of patients with greater care needs may undergo more extensive training or seek out additional resources to enhance their caregiving skills (26, 27).

Furthermore, the correlation analysis and path analysis results provide additional insights into the complex interrelationships between caregiver KAP constructs. The positive correlations between knowledge, attitudes, and practices suggest a cohesive framework wherein caregivers with higher knowledge levels exhibit more positive attitudes and engage in proactive caregiving practices. These findings are consistent with the tenets of the theory of planned behavior, which posits that attitudes, subjective norms, and perceived behavioral control influence behavioral intentions and actions (28). Moreover, the path analysis elucidates the direct effects of knowledge and attitudes on caregiving practices, highlighting potential pathways for intervention targeting caregiver education and attitude enhancement. These results underscore the importance of multifaceted interventions addressing knowledge gaps and fostering positive caregiving attitudes to promote optimal caregiving practices.

Among the knowledge items assessed, the highest scoring item pertained to the recognition of the sequelae of ischemic stroke, including speech impairment, limb paralysis, and others. This high level of awareness is crucial as it indicates caregivers’ understanding of the potential challenges faced by patients’ post-stroke. Conversely, the lowest scoring item related to the recognition of common treatment modalities for ischemic stroke, such as medication, thrombolysis, and thrombectomy. To address the identified gaps in caregivers’ knowledge regarding ischemic stroke treatment modalities, targeted educational interventions are warranted. First and foremost, organizing workshops led by healthcare professionals would offer caregivers opportunities to deepen their understanding of common treatment approaches, including medication, thrombolysis, and thrombectomy. These workshops should be designed to be interactive, allowing caregivers to engage in discussions and seek clarification on complex topics. Additionally, the development of accessible educational materials, such as pamphlets or online resources, would provide caregivers with readily available information in various formats and languages (29). Dissemination of these materials through multiple channels, including hospitals and online platforms, would ensure broad accessibility. Moreover, establishing peer support networks could foster knowledge-sharing among caregivers, enabling them to exchange experiences and insights related to stroke caregiving (30, 31).

In terms of attitudes toward caregiving, caregivers overwhelmingly recognized their crucial role in the patient’s disease rehabilitation process. This positive attitude underscores caregivers’ sense of responsibility and commitment to supporting patients through their recovery journey. Conversely, a notable proportion of caregivers expressed neutral or negative attitudes toward actively seeking knowledge and participating in in-hospital education related to home care for ischemic stroke patients, indicating potential barriers to engagement with educational resources. Efforts to enhance caregivers’ attitudes toward stroke caregiving can be achieved through various strategies. Enhancing caregivers’ attitudes toward stroke caregiving involves promoting in-hospital education sessions dedicated to home care for ischemic stroke patients. Incentivizing attendance can further motivate engagement with educational opportunities. Moreover, developing user-friendly online learning platforms tailored to stroke caregiving offers convenient access to educational resources. Encouraging participation in online courses and forums facilitates knowledge acquisition and promotes positive attitudes toward continuous learning. Additionally, implementing peer mentorship programs where experienced caregivers serve as mentors fosters a supportive caregiving community, providing emotional support and practical guidance to newcomers (32, 33).

In terms of caregiving practices, caregivers demonstrated a high frequency of engagement in activities related to assisting patients with personal hygiene and nutritional support, indicating a strong commitment to meeting patients’ basic needs. However, the frequency of providing emotional support and conducting psychological interventions was comparatively lower, suggesting a potential gap in addressing patients’ psychological well-being during home care. To enhance caregivers’ caregiving practices, targeted interventions focusing on skills training, multidisciplinary collaboration, and home visitation programs are essential. Improving caregivers’ caregiving practices necessitates targeted interventions focusing on skills training, multidisciplinary collaboration, and home visitation programs. Skills training workshops equip caregivers with effective communication techniques and psychological support strategies through role-playing exercises and case studies. Facilitating collaboration between caregivers and multidisciplinary care teams ensures comprehensive stroke patient care through open communication and information-sharing. Furthermore, implementing home visitation programs staffed by trained healthcare professionals or volunteers offers personalized support and guidance to caregivers in real-life settings, promoting optimal care for stroke patients (34, 35).

This study had several limitations. Firstly, the reliance on self-reported data via a web-based questionnaire may introduce response bias and limit the depth of information obtained, such as the disability status of the patient should be assessed by the mRS score which will make it more objective. Secondly, the cross-sectional design prevents the establishment of causality and may not capture changes in KAP over time. However, despite these limitations, the strengths of this paper lie in its comprehensive assessment of family caregivers’ KAP toward home-based care for patients with cerebral infarction, utilizing multivariate logistic regression and path analysis to provide valuable insights into factors influencing caregiving practices. Additionally, the large sample size enhances the robustness of the study’s findings, laying a foundation for future research and intervention development in this critical area of healthcare.

Therefore, implementing targeted educational interventions is crucial to enhance their knowledge base, thereby positively influencing their attitudes and practices in caring for these patients at home. Future efforts should include a follow-up study to assess the impact of these interventions on KAP, enabling a comprehensive evaluation of how educational measures translate into practical caregiving improvements.

## Data availability statement

The original contributions presented in the study are included in the article/Supplementary material, further inquiries can be directed to the corresponding authors.

## Ethics statement

The studies involving humans were approved by Medical Ethics Committee of Yancheng Third People's Hospital Medical Ethics Committee (No. 2023-61). The studies were conducted in accordance with the local legislation and institutional requirements. The participants provided their written informed consent to participate in this study.

## Author contributions

ZC: Data curation, Formal analysis, Investigation, Writing – original draft, Writing – review & editing. XZ: Data curation, Formal analysis, Investigation, Writing – original draft, Writing – review & editing. LJ: Data curation, Formal analysis, Investigation, Writing – original draft, Writing – review & editing. CS: Conceptualization, Data curation, Formal analysis, Investigation, Writing – review & editing. SW: Conceptualization, Data curation, Formal analysis, Investigation, Writing – review & editing. HZ: Data curation, Formal analysis, Investigation, Writing – review & editing. JL: Data curation, Formal analysis, Investigation, Writing – review & editing. XM: Data curation, Formal analysis, Investigation, Writing – review & editing. JY: Data curation, Formal analysis, Investigation, Writing – review & editing.

## Footnotes

^1^ http://www.sojump.com
