# Supplementary material for: Knowledge, attitudes, and practices of family caregivers for patients with cerebral infarction toward home-based care
Source: Front Public Health. 2024 Aug 20;12:1436423. doi: 10.3389/fpubh.2024.1436423 (PMC11368753; doi:10.3389/fpubh.2024.1436423)
Supplement: Supplementary file 1 [file Table_1.DOC]

**Table S1 Pearson’s Analysis**

|  | Knowledge | Attitudes | Practices |
| --- | --- | --- | --- |
| Knowledge | 1 |  |  |
| Attitudes | 0.339(P<0.001) | 1 |  |
| Practices | 0.546(P<0.001) | 0.416(P<0.001) | 1 |

**Table S2. Percentage of sufficient knowledge, favorable attitudes, and proactive practices**

| Cutoff 70% | range | n | % |
| --- | --- | --- | --- |
| Knowledge | (0, 6.3] | 303 | 39.82 |
|  | (6.3, 9] | 458 | 60.18 |
| Attitudes | (9, 31.5] | 193 | 25.36 |
|  | (31.5, 45] | 568 | 74.64 |
| Practices | (8, 28] | 216 | 28.38 |
|  | (28, 40] | 545 | 71.62 |

**Table S3. Multivariate analysis of knowledge**

|  | **Univariate analysis** | | **Multivariate analysis** | |
| --- | --- | --- | --- | --- |
|  | **OR (95%CI)** | **P** | **OR (95%CI)** | **P** |
| **Gender** |  |  |  |  |
| Male | ref. |  |  |  |
| Female | 1.130(0.841,1.518) | 0.418 |  |  |
| **Age** |  |  |  |  |
| 18-30 years old | ref. |  |  |  |
| 31-50 years old | 2.014(0.726,5.584) | 0.179 |  |  |
| 51-70 years old | 1.606(0.591,4.365) | 0.353 |  |  |
| ≥71 years old | 0.762(0.267,2.176) | 0.612 |  |  |
| **Residence** |  |  |  |  |
| Rural | ref. |  | ref. |  |
| Urban | 1.444(1.075,1.940) | 0.015 | 1.335(0.922,1.932) | 0.126 |
| **Marital status** |  |  |  |  |
| Unmarried/Divorced/Other | 0.703(0.326,1.518) | 0.370 |  |  |
| Married | ref. |  |  |  |
| **Education** |  |  |  |  |
| Junior high school and below | ref. |  | ref. |  |
| High school and technical school | 1.955(1.386,2.757) | <0.001 | 1.705(1.124,2.586) | 0.012 |
| College and above | 1.777(1.049,3.010) | 0.033 | 1.397(0.702,2.778) | 0.341 |
| **Average Monthly Household Income (CNY)** |  |  |  |  |
| <2000 | ref. |  | ref. |  |
| 2000-5000 | 1.075(0.719,1.608) | 0.725 | 0.864(0.523,1.428) | 0.569 |
| 5000-10000 | 1.386(0.868,2.214) | 0.172 | 0.852(0.457,1.586) | 0.613 |
| >10000 | 6.943(1.544,31.221) | 0.012 | 5.830(1.060,32.072) | 0.043 |
| **Occupation** |  |  |  |  |
| Formal employment/part-time/self-employment | ref. |  | ref. |  |
| Unemployed | 0.995(0.457,2.165) | 0.990 | 0.733(0.300,1.790) | 0.495 |
| Retired | 1.180(0.825,1.688) | 0.365 | 1.361(0.877,2.112) | 0.170 |
| Other | 0.520(0.353,0.767) | 0.001 | 0.642(0.388,1.062) | 0.084 |
| **Relationship** |  |  |  |  |
| Spouse of the patient | ref. |  | ref. |  |
| Children of the patient | 1.243(0.923,1.676) | 0.153 | 1.085(0.741,1.588) | 0.676 |
| Other family relationship | 2.226(0.969,5.116) | 0.059 | 2.160(0.831,5.611) | 0.114 |
| Employment/other | 4.646(1.024,21.073) | 0.046 | 7.856(1.436,42.986) | 0.017 |
| **Living with the patient** |  |  |  |  |
| Yes | 1.033(0.748,1.426) |  |  |  |
| No | ref. |  |  |  |
| **Patient's Gender** |  |  |  |  |
| Male | ref. |  |  |  |
| Female | 0.906(0.675,1.216) | 0.510 |  |  |
| **Patient's Age** |  |  |  |  |
| 31-50 years old | ref. |  |  |  |
| 51-70 years old | 0.567(0.244,1.315) | 0.186 |  |  |
| ≥71 years old | 0.518(0.225,1.189) | 0.121 |  |  |
| **Patient's Smoking Habit** |  |  |  |  |
| Yes | 0.836(0.616,1.137) | 0.254 |  |  |
| No | ref. |  |  |  |
| **Patient's Drinking Habit** |  |  |  |  |
| Yes | 0.892(0.639,1.244) | 0.500 |  |  |
| No | ref. |  |  |  |
| **Disability status of the patient** |  |  |  |  |
| Yes | 2.147(1.583,2.912) | <0.001 | 3.580(2.358,5.436) | <0.001 |
| No | ref. |  | ref. |  |
| **Does the patient prefer a high-salt/high-fat diet?** |  |  |  |  |
| Yes | 0.953(0.711,1.276) | 0.745 |  |  |
| No | ref. |  |  |  |
| **Patient’s Exercise Frequency** |  |  |  |  |
| 1~2 times a week | 0.458(0.252,0.834) | 0.011 | 0.665(0.340,1.302) | 0.234 |
| 3~4 times a week | 1.621(0.934,2.815) | 0.086 | 2.366(1.279,4.379) | 0.006 |
| 5~6 times a week | 3.126(1.830,5.341) | <0.001 | 6.364(3.384,11.968) | <0.001 |
| Every day | 1.010(0.642,1.588) | 0.967 | 2.352(1.307,4.233) | 0.004 |
| Never | ref. |  | ref. |  |
| **Duration of the patient's illness** |  |  |  |  |
| Less than one month | ref. |  | ref. |  |
| 1~6 months | 1.110(0.683,1.802) | 0.674 | 1.098(0.644,1.873) | 0.732 |
| 6~12 months | 0.753(0.490,1.159) | 0.197 | 0.792(0.492,1.276) | 0.338 |
| 12~18 months | 0.815(0.351,1.893) | 0.634 | 0.843(0.331,2.149) | 0.721 |
| 18~24 months | 1.455(0.752,2.817) | 0.266 | 2.084(0.993,4.373) | 0.052 |
| ＞24 months | 0.597(0.406,0.877) | 0.009 | 0.735(0.468,1.154) | 0.181 |

**Table S4 Multivariate analysis of attitudes**

|  | **Univariate analysis** | | **Multivariate analysis** | |
| --- | --- | --- | --- | --- |
|  | **OR (95%CI)** | **P** | **OR (95%CI)** | **P** |
| **Knowledge score** | 1.485(1.346,1.639) | <0.001 | 1.420(1.274,1.583) | <0.001 |
| **Gender** |  |  |  |  |
| Male | ref. |  |  |  |
| Female | 0.766(0.546,1.074) | 0.122 |  |  |
| **Age** |  |  |  |  |
| 18-30 years old | ref. |  |  |  |
| 31-50 years old | 0.477(0.105,2.171) | 0.338 |  |  |
| 51-70 years old | 0.465(0.104,2.082) | 0.317 |  |  |
| ≥71 years old | 0.226(0.049,1.043) | 0.057 |  |  |
| **Residence** |  |  |  |  |
| Rural | ref. |  |  |  |
| Urban | 1.346(0.967,1.871) | 0.078 |  |  |
| **Marital status** |  |  |  |  |
| Unmarried/Divorced/Other | 0.970(0.404,2.330) | 0.945 |  |  |
| Married | ref. |  |  |  |
| **Education** |  |  |  |  |
| Junior high school and below | ref. |  | ref. |  |
| High school and technical school | 2.238(1.489,3.363) | <0.001 | 1.680(1.061,2.661) | 0.027 |
| College and above | 3.168(1.535,6.541) | 0.002 | 2.594(1.084,6.208) | 0.032 |
| **Average Monthly Household Income (CNY)** |  |  |  |  |
| <2000 | ref. |  | ref. |  |
| 2000-5000 | 1.456(0.948,2.235) | 0.086 | 1.572(0.924,2.675) | 0.096 |
| 5000-10000 | 2.184(1.290,3.700) | 0.004 | 1.602(0.811,3.162) | 0.175 |
| >10000 | 2.902(0.805,10.465) | 0.103 | 0.617(0.137,2.778) | 0.529 |
| **Occupation** |  |  |  |  |
| Formal employment/part-time/self-employment | ref. |  | ref. |  |
| Unemployed | 4.113(0.960,17.623) | 0.057 | 3.628(0.798,16.496) | 0.095 |
| Retired | 0.972(0.650,1.454) | 0.891 | 1.059(0.675,1.659) | 0.803 |
| Other | 0.521(0.345,0.789) | 0.002 | 0.873(0.516,1.475) | 0.611 |
| **Relationship** |  |  |  |  |
| Spouse of the patient | ref. |  |  |  |
| Children of the patient | 1.386(0.989,1.943) | 0.058 |  |  |
| Other family relationship | 1.374(0.574,3.292) | 0.476 |  |  |
| Employment/other | 1.002(0.307,3.270) | 0.997 |  |  |
| **Living with the patient** |  |  |  |  |
| Yes | 0.724(0.495,1.058) | 0.095 |  |  |
| No | ref. |  |  |  |
| **Patient's Gender** |  |  |  |  |
| Male | ref. |  | ref. |  |
| Female | 1.489(1.060,2.091) | 0.022 | 1.651(1.136,2.398) | 0.009 |
| **Patient's Age** |  |  |  |  |
| 31-50 years old | ref. |  |  |  |
| 51-70 years old | 1.594(0.696,3.648) | 0.270 |  |  |
| ≥71 years old | 1.104(0.492,2.478) | 0.811 |  |  |
| **Patient's Smoking Habit** |  |  |  |  |
| Yes | 0.718(0.511,1.008) | 0.056 |  |  |
| No | ref. |  |  |  |
| **Patient's Drinking Habit** |  |  |  |  |
| Yes | 0.818(0.566,1.183) | 0.286 |  |  |
| No | ref. |  |  |  |
| **Disability status of the patient** |  |  |  |  |
| Yes | 1.546(1.100,2.172) | 0.012 | 1.304(0.893,1.904) | 0.170 |
| No | ref. |  | ref. |  |
| **Does the patient prefer a high-salt/high-fat diet?** |  |  |  |  |
| Yes | 0.809(0.581,1.127) | 0.211 |  |  |
| No | ref. |  |  |  |
| **Patient’s Exercise Frequency** |  |  |  |  |
| 1~2 times a week | 0.954(0.498,1.828) | 0.887 |  |  |
| 3~4 times a week | 1.266(0.683,2.347) | 0.454 |  |  |
| 5~6 times a week | 0.985(0.570,1.704) | 0.958 |  |  |
| Every day | 1.246(0.747,2.081) | 0.400 |  |  |
| Never | ref. |  |  |  |
| **Duration of the patient's illness** |  |  |  |  |
| Less than one month | ref. |  | ref. |  |
| 1~6 months | 2.231(1.156,4.306) | 0.017 | 2.169(1.091,4.312) | 0.027 |
| 6~12 months | 0.529(0.337,0.830) | 0.006 | 0.513(0.315,0.835) | 0.007 |
| 12~18 months | 2.314(0.672,7.965) | 0.183 | 2.532(0.715,8.973) | 0.150 |
| 18~24 months | 1.142(0.557,2.340) | 0.717 | 1.191(0.555,2.555) | 0.653 |
| ＞24 months | 0.899(0.584,1.386) | 0.631 | 1.171(0.716,1.915) | 0.529 |

**Table S5 Multivariate analysis of practices**

|  | **Univariate analysis** | | **Multivariate analysis** | |
| --- | --- | --- | --- | --- |
|  | **OR (95%CI)** | **P** | **OR (95%CI)** | **P** |
| **Knowledge score** | 1.999(1.772,2.256) | <0.001 | 1.756(1.541,2.001) | <0.001 |
| **attitudes score** | 1.299(1.202,1.404) | <0.001 | 1.203(1.098,1.318) | <0.001 |
| **Gender** |  |  |  |  |
| Male | ref. |  |  |  |
| Female | 1.257(0.914,1.729) | 0.160 |  |  |
| **Age** |  |  |  |  |
| 18-30 years old | ref. |  |  |  |
| 31-50 years old | 0.915(0.306,2.734) | 0.874 |  |  |
| 51-70 years old | 1.520(0.515,4.482) | 0.448 |  |  |
| ≥71 years old | 0.719(0.234,2.212) | 0.565 |  |  |
| **Residence** |  |  |  |  |
| Rural | ref. |  |  |  |
| Urban | 1.310(0.953,1.803) | 0.097 |  |  |
| **Marital status** |  |  |  |  |
| Unmarried/Divorced/Other | 0.786(0.347,1.777) | 0.562 |  |  |
| Married | ref. |  |  |  |
| **Education** |  |  |  |  |
| Junior high school and below | ref. |  |  |  |
| High school and technical school | 1.802(1.231,2.637) | 0.002 | 1.394(0.867,2.241) | 0.171 |
| College and above | 1.552(0.872,2.763) | 0.135 | 1.099(0.534,2.262) | 0.798 |
| **Average Monthly Household Income (CNY)** |  |  |  |  |
| <2000 | ref. |  |  |  |
| 2000-5000 | 0.980(0.632,1.520) | 0.928 |  |  |
| 5000-10000 | 1.055(0.636,1.752) | 0.835 |  |  |
| >10000 | 7.773(1.003,60.253) | 0.050 |  |  |
| **Occupation** |  |  |  |  |
| Formal employment/part-time/self-employment | ref. |  | ref. |  |
| Unemployed | 2.513(0.855,7.385) | 0.094 | 2.102(0.614,7.188) | 0.237 |
| Retired | 1.440(0.963,2.154) | 0.076 | 1.255(0.770,2.045) | 0.362 |
| Other | 0.610(0.408,0.912) | 0.016 | 0.900(0.532,1.520) | 0.693 |
| **Relationship** |  |  |  |  |
| Spouse of the patient | ref. |  |  |  |
| Children of the patient | 0.944(0.682,1.307) | 0.728 |  |  |
| Other family relationship | 0.698(0.322,1.512) | 0.362 |  |  |
| Employment/other | 2.304(0.506,10.485) | 0.280 |  |  |
| **Living with the patient** |  |  |  |  |
| Yes | 1.522(1.083,2.140) | 0.016 | 2.046(1.314,3.187) | 0.002 |
| No | ref. |  | ref. |  |
| **Patient's Gender** |  |  |  |  |
| Male | ref. |  |  |  |
| Female | 1.215(0.880,1.677) | 0.237 |  |  |
| **Patient's Age** |  |  |  |  |
| 31-50 years old | ref. |  |  |  |
| 51-70 years old | 1.487(0.667,3.317) | 0.333 |  |  |
| ≥71 years old | 1.150(0.524,2.525) | 0.728 |  |  |
| **Patient's Smoking Habit** |  |  |  |  |
| Yes | 0.769(0.553,1.068) | 0.117 |  |  |
| No | ref. |  |  |  |
| **Patient's Drinking Habit** |  |  |  |  |
| Yes | 0.909(0.635,1.304) | 0.605 |  |  |
| No | ref. |  |  |  |
| **Disability status of the patient** |  |  |  |  |
| Yes | 1.328(0.960,1.836) | 0.086 |  |  |
| No | ref. |  |  |  |
| **Does the patient prefer a high-salt/high-fat diet?** |  |  |  |  |
| Yes | 1.091(0.795,1.498) | 0.588 |  |  |
| No | ref. |  |  |  |
| **Patient’s Exercise Frequency** |  |  |  |  |
| 1~2 times a week | 0.858(0.478,1.543) | 0.610 | 1.334(0.645,2.757) | 0.437 |
| 3~4 times a week | 2.813(1.548,5.112) | 0.001 | 2.792(1.383,5.637) | 0.004 |
| 5~6 times a week | 6.961(3.686,13.146) | <0.001 | 5.437(2.635,11.22) | <0.001 |
| Every day | 1.623(1.022,2.579) | 0.040 | 1.505(0.872,2.597) | 0.142 |
| Never | ref. |  | ref. |  |
| **Duration of the patient's illness** |  |  |  |  |
| Less than one month | ref. |  | ref. |  |
| 1~6 months | 1.152(0.666,1.994) | 0.612 | 0.853(0.447,1.628) | 0.629 |
| 6~12 months | 0.933(0.576,1.511) | 0.776 | 1.090(0.610,1.946) | 0.772 |
| 12~18 months | 1.277(0.462,3.532) | 0.637 | 1.206(0.383,3.799) | 0.748 |
| 18~24 months | 1.036(0.515,2.084) | 0.920 | 0.879(0.383,2.018) | 0.762 |
| ＞24 months | 0.467(0.312,0.698) | <0.001 | 0.581(0.347,0.973) | 0.039 |

**Table S6 Test results of the hypothesis**

|  |  |  | Estimate | P |
| --- | --- | --- | --- | --- |
| Attitudes | <--- | Knowledge | 0.885 | <0.001 |
| Practices | <--- | Attitudes | 0.838 | <0.001 |
| Practices | <--- | Knowledge | 1.295 | <0.001 |

**Table S7 Model fitness indices for the KAP structural equation model**

| Indicators | Reference | Actual |
| --- | --- | --- |
| CMIN/DF | 1-3: Excellent, 3-5: Good | 3.359 |
| RMSEA | <0.08: Good | 0.065 |
| IFI | >0.8: Good | 0.848 |
| TLI | >0.8: Good | 0.818 |
| CFI | >0.8: Good | 0.846 |
